# Null hypothesis significance testing vs. Bayesian inference using generalized linear mixed models with binary outcomes: a case study under practical design constraints

**DOI:** 10.3389/fpsyg.2026.1770212

**Published:** 2026-04-16

**Authors:** Stefano Dalla Bona, Andrea Spoto, Marta Caserotti, Lorella Lotto, Michela Sarlo, Nicola Cellini, Simone Cutini, Giovanni Bruno

**Affiliations:** 1 Department of General Psychology, Universita Degli Studi di Padova, Padova, Italy; 2 Department of Developmental Psychology and Socialisation, Università degli Studi di Padova, Padova, Italy; 3 Department of Communication Sciences, Humanities and International Studies, Universita degli Studi di Urbino Carlo Bo, Urbino, Italy

**Keywords:** Bayes Factor, Bayesian analysis, moral dilemmas, NHST analysis, underpowered designs

## Abstract

Empirical investigation requires dealing with fundamental uncertainty. In experimental psychology, research questions are often addressed using Null Hypothesis Significance Testing (NHST), an approach rooted in the frequentist statistical tradition. In scenarios that do not consent to reject the null hypothesis using the NHST paradigm (i.e., results are non-significant), researchers may be tempted to reframe their analysis in the Bayesian framework, either as a complementary alternative or alongside the original NHST approach. In fact, the Bayesian approach is gaining increasing appeal in the social sciences as an alternative to the frequentist NHST framework, and Bayesian methods for hypothesis testing (i.e., the Bayes Factor) can be used to help determine whether a failure to reject the null hypothesis reflects merely insufficient evidence for the alternative hypothesis or provides affirmative evidence for the (point) null hypothesis. Nevertheless, using the two approaches interchangeably carries the risk of conceptual confusion, as NHST and Bayesian frameworks address different inferential questions. This study provides an empirical, real-world opportunity to examine how NHST and Bayesian methods can be applied to the same hypothesis test when using Generalized Linear Mixed Models with a Binary Outcome. Importantly, this application incorporates common experimental constraints into the design-analysis planning, defining a reachable, realistic albeit underpowered sample size, assuming the classical 0.80 power threshold. This research report provides a valuable opportunity to examine how Bayesian and NHST approaches potentially differ in their workflow, performance, and inferential interpretation under realistic experimental conditions.

## 1 Introduction

As with all empirical testing, the investigation of psychological phenomena inevitably involves dealing with fundamental uncertainty. In experimental psychology, research questions are often addressed using Null Hypothesis Significance Testing (NHST), an approach rooted in the frequentist statistical tradition. This framework draws from Fisher’s approach to significance testing (Fisher, 1971/1935; Fisher et al., 1990) and from the Neyman and Pearson approach of statistical hypothesis testing (Neyman and Pearson, 1928), and the combination of these distinct approaches (Lindquist, 1940) resulted in the hybrid method for statistical inference widely used today (Gigerenzer, 2004; Perezgonzalez, 2015). In scenarios where the test statistics calculated from observed data do not consent to reject the null hypothesis using the NHST paradigm (i.e., results are non-significant), researchers may be tempted to reframe and re-conduct their analysis in the Bayesian framework, either as a complementary alternative or presented alongside the original NHST results (Tendeiro and Kiers, 2019). This practice can be motivated by the fact that the Bayesian approach is gaining increasing appeal in the social sciences and in sciences more generally (e.g., in educational research; König and van de Schoot, 2018; in ecology; Banner et al., 2020) as an alternative to the frequentist NHST framework (Myung and Pitt, 1997; van de Schoot et al., 2017). Furthermore, certain Bayesian methods for hypothesis testing (i.e., the Bayes Factor) can be used to help determine whether a failure to reject the null hypothesis reflects merely insufficient evidence or instead provides affirmative evidence for the (point) null hypothesis (Dienes, 2014), and clarify whether non-significant findings stem from inadequate evidential strength due to underpowered samples (e.g., Etz and Vandekerckhove, 2016). Bayesian alternatives to NHST have been widely illustrated and discussed in the literature, particularly in how they address well-known shortcomings of NHST, such as the overreliance on *p*-values and the use of discretional significance thresholds (Curran, 2009; Ioannidis, 2016), and in presenting potential statistical alternatives to the classical frequentist approach (Kelter, 2020; Kruschke, 2011; Ortega and Navarrete, 2017; Tendeiro and Kiers, 2019). Nevertheless, adopting the strategy of use Bayesian methods after the NHST approach yielded unsatisfactory information, carries the risk of conceptual confusion, as NHST and Bayesian frameworks address fundamentally different inferential questions: the former evaluates the probability of observing the data assuming an hypothesis in which distributional characteristics of a certain statistical test value are known (i.e., typically a point-null hypothesis), while the latter assesses the probability of a hypothesis (i.e., a statistical model) given the observed data and the priors (Kruschke, 2011; Nathoo and Masson, 2016). Compared to the NHST framework, Bayesian analysis enables a shift from a binary mode of reasoning (i.e., “Is there evidence against the null hypothesis?”) to a more nuanced interpretation based on the magnitude of evidence - a “thermometer” for the intensity of evidence (Wagenmakers et al., 2018) - and likelihood, potentially offering a richer depiction of the phenomenon under investigation (Ortega and Navarrete, 2017), though based on a different approach to evaluating uncertainty and evidence (i.e., “How plausible is each hypothesis given the data?”). Following this logic, it is advisable to acknowledge their conceptual differences before selecting an approach and/or before adopting a comparative perspective to answer a research question. In this work we aim to analyze the same data through both statistical lenses to uncover how each framework interprets - similarly and differently - ambiguous or nonsignificant findings. This approach can highlight the strengths and limitations of both methods in hypothesis testing, specifically when hierarchical models are involved (e.g., Generalized Linear Mixed Models, GLMMs), and fosters more punctual inferences grounded in the assumptions of each approach (Ortega and Navarrete, 2017; Tendeiro and Kiers, 2019). A few notable examples of such comparative approaches are available in the literature (e.g., Nathoo and Masson, 2016; Flores et al., 2022).

This study provides an empirical, real-world opportunity to examine how NHST and Bayesian methods can be applied to the same hypothesis tests within GLMMs involving a binary outcome. It draws on a pre-registered study hosted on the Open Science Framework (OSF), which investigates the role of available Decision Time and the Trial Progression influencing the likelihood of utilitarian outcomes in self-sacrificial moral dilemmas (Registration DOI: 10.17605/OSF.IO/RXK6V). Specifically, we aim to examine the evidence provided by both approaches in cases where no clear effects of the independent variables appear to be present, exploring whether switching from NHST to Bayesian analysis improves the descriptiveness and interpretability of the findings.

Importantly, the focus of the present work is specifically on Hypothesis 1 of the preregistered study, for which the target sample size was determined using an *a priori* power analysis informed by data from Bakker et al. (2016). Importantly, feasibility constraints were explicitly considered when defining target sample size. In practice, factors such as limited personnel and restricted funding can substantially impede large study power, especially when modeling a binomial distribution, whose limited informativeness further reduces statistical efficiency. This application acknowledges these constraints by incorporating them directly into the design-analysis planning, conducting a power-analysis simulation that targeted a power of 0.60, while maintaining a typical Type I error rate *α* = 0.05. Although the fixed sample size introduces constraints, it also provides a valuable opportunity to examine how Bayesian and NHST approaches differ in their workflow, performance, and inferential interpretation under realistic conditions of limited design flexibility.

## 2 Methods

### 2.1 Aims and procedure

The reference project aimed at investigating tendencies toward utilitarianism in the context of moral reasoning applied to autonomous vehicles’ (AVs) behavior (e.g., Bonnefon et al., 2016). The investigation aimed at understanding how contextual factors may intervene in favor of utilitarian maneuvers when AVs have to face trolley-like situations with sacrificial outcomes (Bruno et al., 2023a, 2023b). The project had three hypotheses, albeit the power analysis was based on the first one (H1), evaluating the interactive impact of available decision time and of trial progression on the utilitarian moral response. For additional information on the theoretical background, please refer to the OSF pre-registered project.

Throughout the experiment, participants were required to answer three AV moral dilemmas presented sequentially in a textual form (Bruno et al., 2023b). In each dilemma, the AV had to face a critical decision: in one case the maneuver could result either in the sacrifice of the AV passenger (utilitarian choice) or in the sacrifice of three individuals crossing the road (non-utilitarian choice). Participants were required to assume the AV passenger perspective, thus being directly involved in the final outcome. The sample was randomly divided in three groups, characterized by a different amount of time to decide about the AV maneuver after reading the scenario and the alternatives: the “Pressure” group (7 s maximum), the “Delay” group (wait at least 60 s before answering), and the “Control” group (no imposed decision time). The task was administered inside a laboratory environment, through a computer desktop and with the use of Qualtrics platform^1^.

An a-priori power analysis simulation was carried out in the R environment to define the appropriate sample size, taking advantage of results from a previous data collection (*N* = 206). Considering the experimental design and hypothesis, the modeling of sample variability as a random intercept, and practical recruitment constraints (no funding and limited personnel), we planned a maximum sample size of 180 individuals, corresponding to a statistical power of 60% for the first hypothesis. The final sample consisted of 185 participants. Of these, 63 were allocated to the Pressure group (30 female), 62 were allocated to the Delay group (30 female) and 60 were allocated to the Control group (30 female). All participants were suitable for analysis, as the vast majority correctly answered all three attention checks (*N* = 183) and only two participants failed one of them.

### 2.2 Model for analysis

In line with the pre-registration, a logistic mixed-effects model was fitted to predict participants’ utilitarian likelihood, coded dichotomously (0 = non-utilitarian choice; 1 = utilitarian choice). The fixed-effect predictors were: (i) Experimental Condition (i.e., available Decision Time), coded as treatment contrasts (setting Control as reference level), and (ii) Trial Progression (i.e., the Dilemmas Order, which refers to the sequence position of the dilemmas—first, second, or third—presented to each participant) coded using Helmert-like contrasts (first vs. second and third dilemmas combined; second vs. third dilemma). Interaction terms between condition and order were included. Participant-level variability was modeled via a random intercept. The model was specified as:


$$\begin{array}{l} \text{logit}[\mathrm{Pr}(y_{\mathrm{ij}}=1)]=\beta_{0}+\beta_{1}\;\text{Dela}y_{\mathrm{ij}}+\beta_{2}\;\text{Pressur}e_{\mathrm{ij}}\\ +\beta_{3}\;\text{Order}1_{\mathrm{ij}}+\beta_{4}\;\text{Order}2_{\mathrm{ij}}+\beta_{5}\;{\left(\text{Delay}\times \text{Order}1\right)}_{\mathrm{ij}}\\ +\beta_{6}\;{\left(\text{Pressure}\times \text{Order}1\right)}_{\mathrm{ij}}+\beta_{7}\;{\left(\text{Delay}\times \text{Order}2\right)}_{\mathrm{ij}}\\ +\beta_{8}\;{\left(\text{Pressure}\times \text{Order}2\right)}_{\mathrm{ij}}+u_{0j}\; \end{array}$$

Data analyses were conducted using R (R Core Team, 2024). For the NHST analysis, the model was fitted with the glmer function from the lme4 package (Bates et al., 2015) and estimated via Maximum Likelihood (ML). For the Bayesian analysis, the model was specified using the brm function from the brms package (Bürkner, 2017, 2018), which closely aligns in code syntax with glmer. The Bayesian models were compiled and estimated using Stan (Stan Development Team, 2025). Additional details regarding the model specification and the statistical metrics employed are provided in the Appendix.

## 3 Results

**Figure 1:**
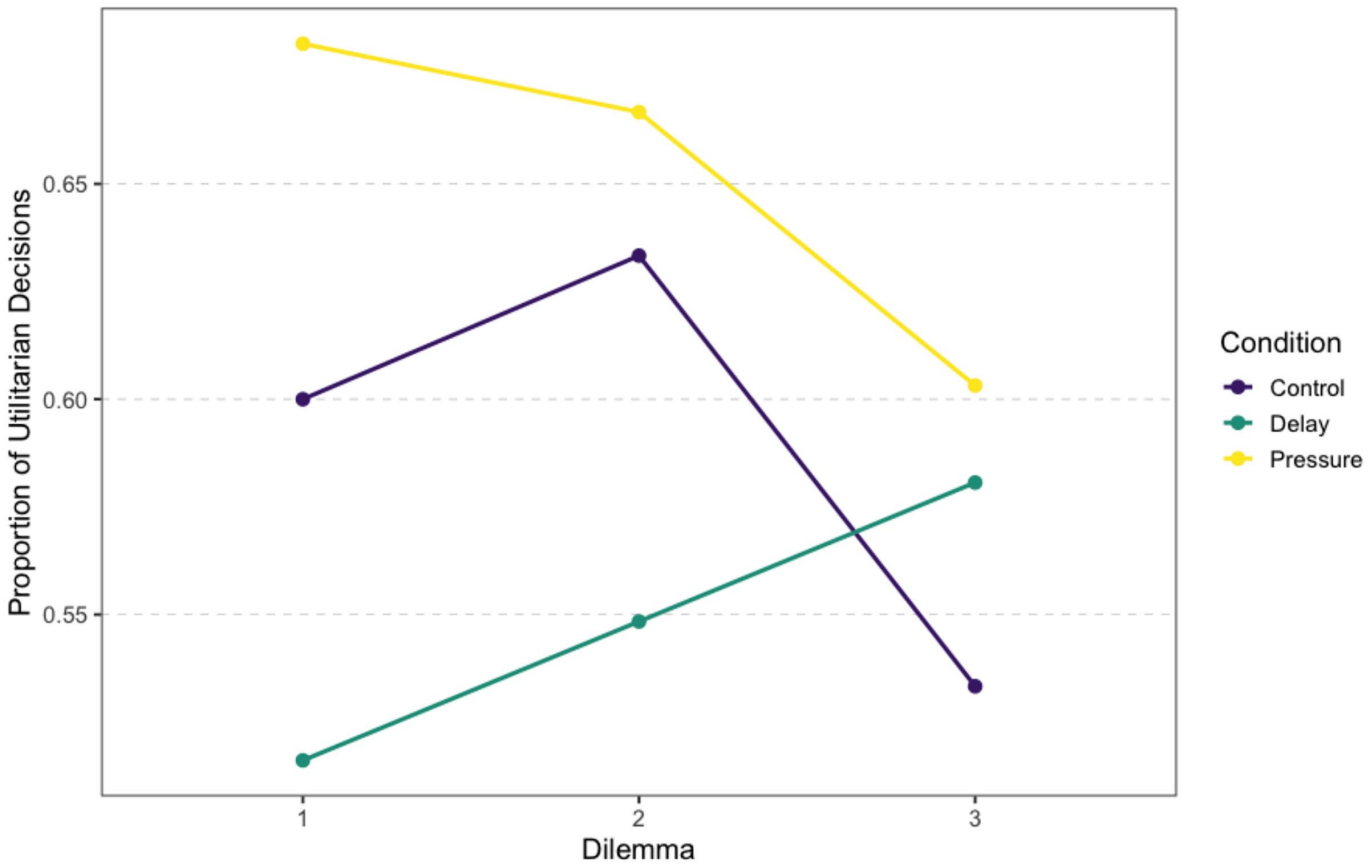
Descriptive results. Proportion of utilitarian decisions across the three dilemmas by experimental condition.

Figure 1 shows utilitarian choice proportions across the three dilemmas. In the Control group, the proportions were 0.60 in the first dilemma, 0.63 in the second, and 0.53 in the third. The Delay group showed proportions of 0.52, 0.55, and 0.58 across the three dilemmas, respectively. The Pressure group showed the highest proportions overall, with 0.68 in the first dilemma, 0.67 in the second, and 0.60 in the third.

### 3.1 Target model

**Figure 2:**
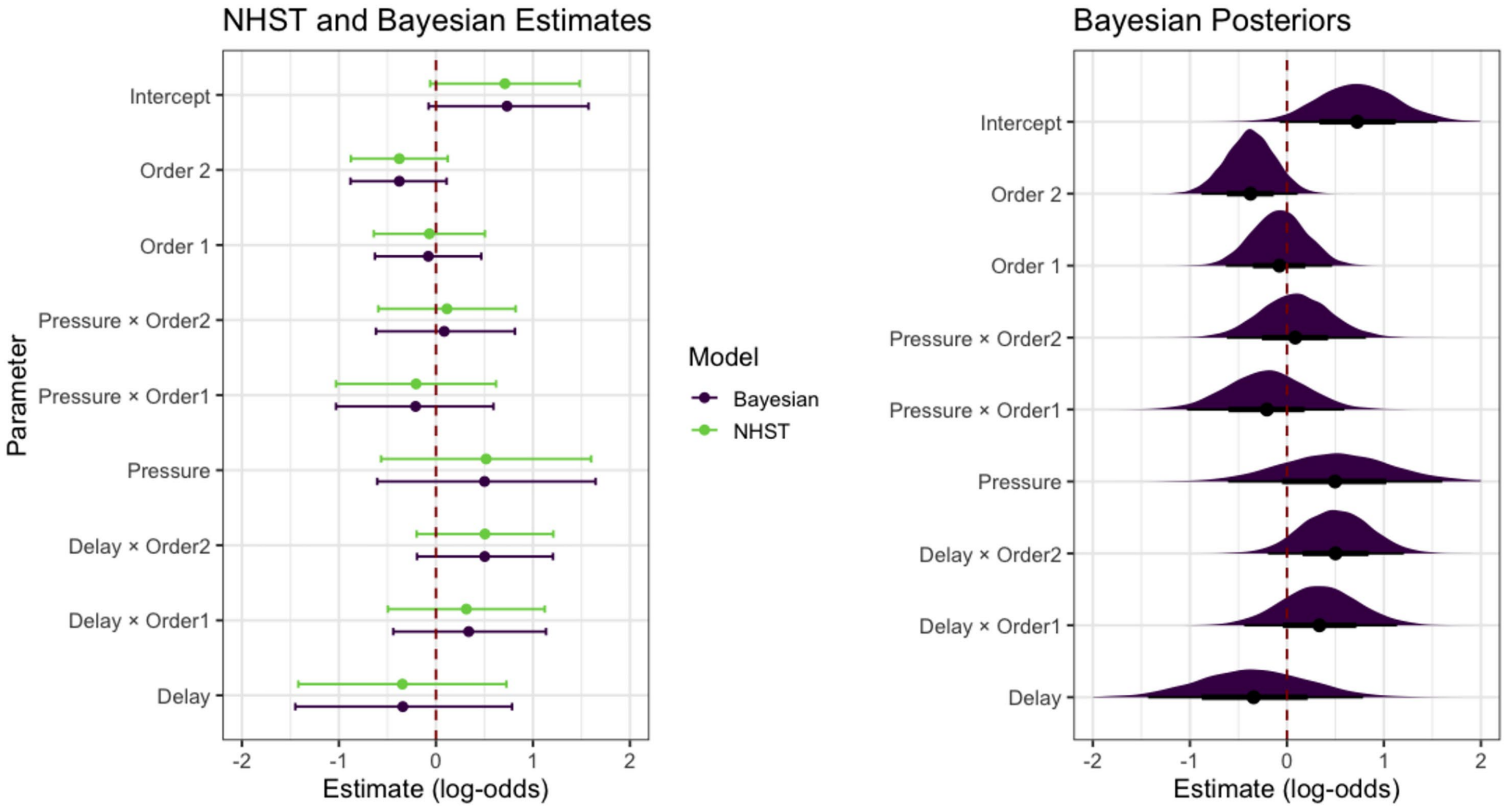
Model estimates. Left panel: fixed parameter estimates with 95% credible intervals (Bayesian) and 95% confidence intervals (NHST). Right panel: posterior distributions of the fixed parameters for the Bayesian model.

We present the target model estimates using both NHST and Bayesian approaches. For each model, parameter point estimates are reported together with confidence intervals for the NHST model and credible intervals for the Bayesian model (see Figure 2, left panel). For NHST estimates, we additionally report *p*-values (i.e., statistical significance), interpreted as the probability of observing the obtained test statistic, or a more extreme one, assuming the null hypothesis is true, whereas for the Bayesian model we report the Probability of Direction (PD, which varies between 50 and 100%; Makowski et al., 2019a), the posterior probability that a parameter is either strictly positive or negative (see Figure 2, right panel), given the data, the model, and the specified prior (Makowski et al., 2019b).

As for the NHST analysis, the logistic mixed-effects model showed substantial overall explanatory power (conditional R^2^ = 0.64), although the variance explained by the fixed effects alone was small (marginal R^2^ = 0.02). The intraclass correlation coefficient extracted from the model (ICC = 0.63) further confirmed that most of the variability in responses was due to differences between individuals rather than experimental predictors. Neither of the Condition contrasts yielded statistically significant effects (Delay: *β* = −0.35, 95% CI [−1.42, 0.73], *p* = 0.53; Pressure: β = 0.52, 95% CI [−0.57, 1.60], *p* = 0.35). The Trial Progression contrasts were likewise non-significant and negative in direction (First dilemma vs. others: β = −0.07, 95% CI [−0.64, 0.50], *p* = 0.82; Second vs. third dilemma: β = −0.38, 95% CI [−0.88, 0.12], *p* = 0.14). All Condition-by-Order parameter interactions were non-significant as well (Delay × First Order: β = 0.31, 95% CI [−0.49, 1.12], *p* = 0.45; Pressure × First Order: β = −0.21, 95% CI [−1.03, 0.62], *p* = 0.63; Delay × Second Order: β = 0.50, 95% CI [−0.20, 1.21], *p* = 0.16; Pressure × Second Order: β = 0.11, 95% CI [−0.59, 0.82], *p* = 0.76). The random intercept SD was 2.38, showing substantial variability across participants. Model performance, diagnostics, prediction checks and Odds Ratio transformations of coefficients are available in the supplementary materials. (available at: https://osf.io/ndcpf).

For the Bayesian analysis, we estimated a Bayesian logistic mixed-effects model using Markov-Chain Monte Carlo (MCMC) sampling (4 chains, 4,000 iterations each, with 1,000 warm-up iterations^2^). The dependent variable and predictors mirrored those of the NHST analysis, with participants included as a random effect. Weakly informative Student-t priors (location = 0, scale = 1.5, df = 7) were placed on the intercept and all regression coefficients (see Gelman et al., 2008; Ghosh et al., 2018 for discussions on priors for logistic regressions). Prior predictive checks and prior sensitivity analyses (based on power scaling; see Kallioinen et al., 2024) supported the adequacy of the specified priors (additional information is available in the supplementary materials). The Bayesian model also showed substantial explanatory adequacy (R^2^ = 0.51, 95% CI [0.45, 0.56]), whereas the proportion of variance attributable to fixed effects alone remained small (marginal R^2^ = 0.06, 95% CI [0.01, 0.12]). Examination of the posterior distributions further supported the conclusion that fixed effects contributed minimally to the model. As for the experimental manipulations, the posterior of the Delay Condition parameter (Median = −0.35, 95% CI [−1.45, 0.78]) showed a low negative PD (PD = 72.39%), whereas the Pressure Condition parameter (Median = 0.50, 95% CI [−0.61, 1.65]) showed a slightly higher, but positive PD (PD = 80.92%). As for the Trial Progression parameters, the First Order parameter (Median = −0.08, 95% CI [−0.63, 0.47]) showed a low negative PD (PD = 61.04%), while the Second Order parameter (Median = −0.38, 95% CI [−0.88, 0.11]) showed a higher negative PD (PD = 93.47%). Interaction parameters showed posterior medians close to zero with moderate uncertainty (Delay × First Order interaction: Median = 0.34, 95% CI [−0.44, 1.13], PD = 80.17%; Pressure × First Order interaction: Median = −0.21, 95% CI [−1.03, 0.59], PD = 69.83%; Delay × Second Order interaction: Median = 0.50, 95% CI [−0.19, 1.21], PD = 92.35%; Pressure × Second Order interaction: Median = 0.09, 95% CI [−0.62, 0.81], PD = 59.34%). All estimated parameters showed convergence (all R̂ ≈ 1.00) and reliable Effective Sample Sizes (ESS values = [4,211, 11,160]). For the equivalence tests, the Region of Practical Equivalence (ROPE) was defined using the standard *π*/3 criterion for logistic models (Kruschke, 2014), and the width of the Credible Intervals, computed as Highest Density Intervals (HDIs), was set to the default 89% (Kruschke, 2014). Across the fixed-effect parameters, the proportion of 89% HDIs falling within the ROPE ranged from 14.50 to 51.85%, indicating inconclusive evidence regarding practical equivalence. Posterior predictive checks, trace plots, p-map values (i.e., the Bayesian analogue of the *p*-value, defined as the ratio of the posterior density at 0 to the density at the Maximum *A Posteriori* estimate, Makowski et al., 2019a; available in the supplementary materials) are reported in the supplementary materials.

We compared the rank-order correspondence between Frequentist *p*-values and several Bayesian summaries (also reported in the supplementary materials) using Kendall’s *τ* (Kendall, 1938). The correlation between p-values and the p-map was perfect (τ = 1). The correlation between p-values and the proportion of 89% HDIs falling within ROPE was also strong (τ = 0.83), suggesting substantial agreement. The correlation between p-values and the PD was strongly negative (τ = −0.94), indicating that predictors with smaller p-values tended to have higher posterior probabilities of a consistent effect direction.

Taken together, while these results show that Bayesian estimates closely align with NHST estimates, the Bayesian approach also provides additional information regarding parameter uncertainty and directional probability. In NHST, a 95% confidence interval for a parameter is an interval produced by a procedure that contains the true value in 95% of repeated samples; however, such intervals cannot be interpreted as the probability of a specific parameter value, nor do they provide a direct measure of the precision of an estimate (see Morey et al., 2016). In contrast, Bayesian credible intervals quantify the posterior probability that the parameter lies within the interval, offering a more direct interpretation of uncertainty. Likewise, whereas the *p*-value expresses the probability of observing a test statistic as equal as or more extreme than the one obtained under the null hypothesis, the PD directly reflects the posterior probability that a parameter is either positive or negative.

### 3.2 Model selection and comparison

**Figure 3:**
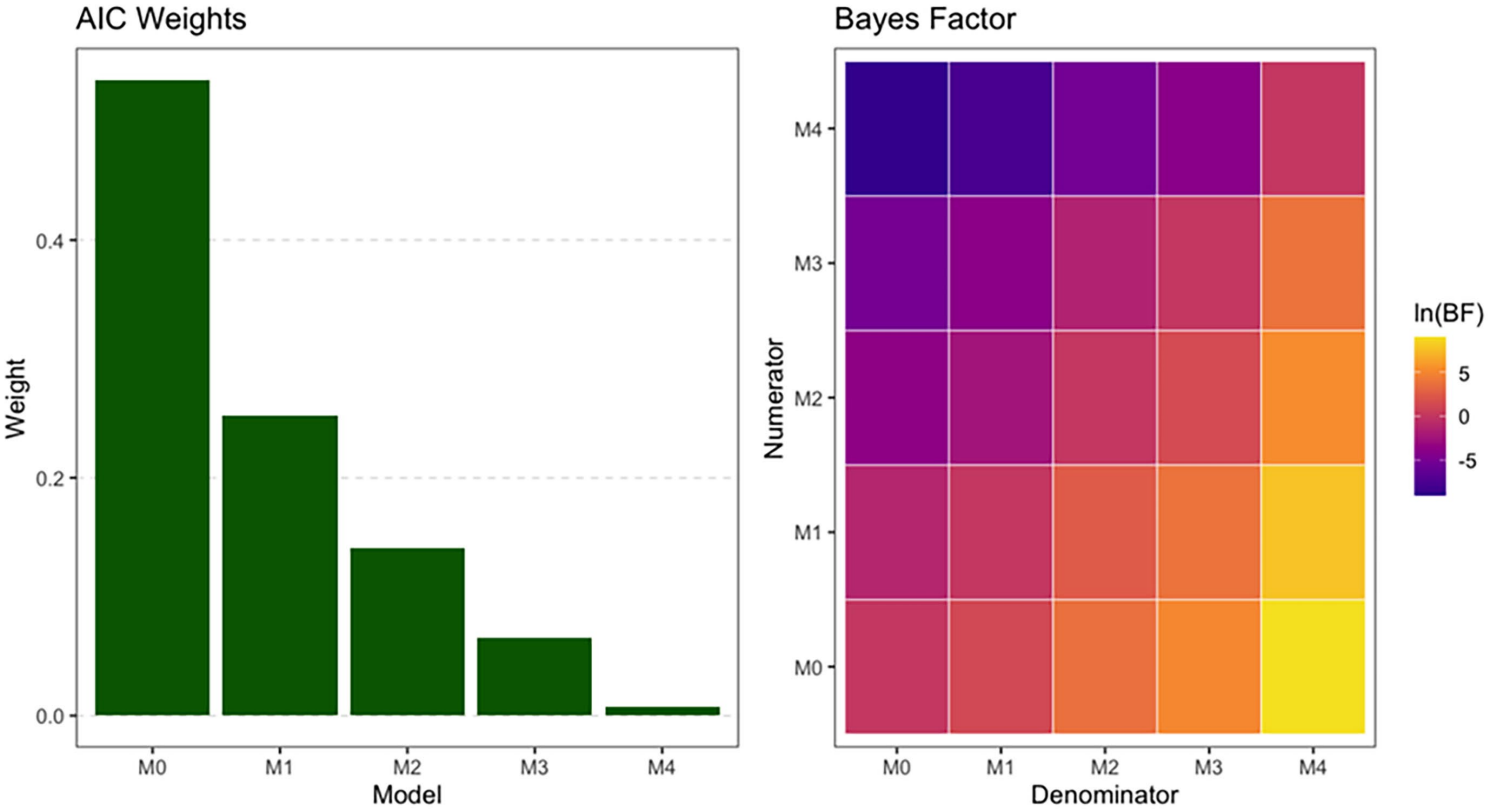
Model selection. AIC transformed into Weights (left panel) for each NHST model and Bayes Factors (expressed on the natural logarithm scale) matrix (right panel) for each Bayesian pairwise model comparisons.

Because the main model did not provide evidence for any interaction term, we conducted a formal model comparison using both frameworks. Within the NHST workflow, model comparison can be performed using Likelihood Ratio Tests (LRT) for comparing nested models and information criteria such as the Akaike Information Criterion (AIC; Akaike, 1974), which are suitable for non-nested models. As our candidate models were not all nested (M0 = intercept only; M1 = Condition; M2 = Trial Progression; M3 = Condition+Trial Progression; M4 = Condition×Trial Progression), we relied on the corrected AIC (AICc) to compare their relative support (see Figure 3, left panel). Notably, the usage of AIC for model selection seems appropriate in this comparative context, as AIC has been shown to rely on the same underlying statistical information as the p-value (Murtaugh, 2014). The simplest mixed-effects model including only an intercept (M0) received the strongest support (AICc = 656.3, weight = 0.535), indicating that adding fixed effects or their interaction did not improve model fit sufficiently to justify the increased complexity. Thus, according to AICc, our data are best explained by the intercept-only mixed-effects model.

For the Bayesian model comparison, we used bridge sampling (Gronau et al., 2017) to estimate the marginal likelihood of each Bayesian logistic mixed-effects model (M0–M4) and computed Bayes Factors^3^ (BFs) for all pairwise comparisons (Student-t – location = 0, scale = 1.5, df = 7 – priors were placed on the intercept and regression coefficients of all models). The resulting BF matrix (see Figure 3, right panel) revealed a clear pattern: the intercept-only model (M0) was strongly preferred over all models including Condition, Dilemma order, or their interaction. Specifically, M0 was favored over M1 (BF = 3.61), M2 (BF = 38.55), M3 (BF = 162.26), and M4 (BF = 8949.40), providing substantial to extreme evidence against the inclusion of additional predictors. Comparisons among the more complex models produced similarly consistent evidence gradients, with model support decreasing as complexity increased and M4 receiving the weakest support overall.

Across both frameworks, the results converge on the same substantive conclusion: there is little to no evidence that the experimental manipulations or dilemma-order variables meaningfully influenced utilitarian decision-making. From the NHST perspective, none of the fixed-effect predictors or interactions were statistically significant. The small marginal R^2^ values indicate that the predictors explain very little variance beyond individual differences. Thus, with respect to the NHST initial guiding question (“Is there evidence against the null hypothesis?”), the answer is no: the data do not provide statistically significant evidence to reject the null hypothesis for any experimental effect. From the Bayesian perspective, the posterior distributions provide some more information, while similarly indicating that the experimental effects are plausibly very small, with posterior medians near zero and substantial uncertainty. Although some parameters showed moderate directional tendencies, the credible intervals were wide and overlapped zero, and ROPE analyses were inconclusive. Regarding the Bayesian initial guiding question (“How plausible is each hypothesis given the data?”), the findings suggest that meaningful effects are not supported by the posterior parameters probabilities. Posterior predictive checks and convergence diagnostics confirm that the weak evidence reflects properties coming from data rather than model inadequacies.

Importantly, the strong correlations between Bayesian indices and NHST *p*-values indicate that both inferential approaches deliver a consistent message. NHST does not identify statistically significant effects, and Bayesian estimation similarly does not provide compelling support for meaningful parameter values. Nonetheless, BF model comparisons offer additional insight by providing comparatively stronger support for the null model over models including experimental predictors, reinforcing the conclusion that the data are more consistent with the absence of an effect than with the presence of a substantial one, even though the BF for the ratio between M0 and M1 was moderate (BF = 3.61).

## 4 Discussion

A direct comparison of the NHST and Bayesian target model estimates revealed a substantial degree of convergence in their practical conclusions: neither approach provided evidence for Decision Time or Trial Progression effects on utilitarian responses. In other words, across both inferential frameworks the observed data offered no compelling support for the existence of an effect, whether expressed as a failure to reject a null hypothesis (NHST) or as low posterior probability for non-zero effects (Bayesian). Bayesian analysis, however, offered an alternative and additive interpretation. Whereas NHST yielded a binary non-rejection of the null, the Bayesian model quantified parameter uncertainty through posterior distributions (conditioned on data, the model and priors). Credible intervals and indices such as the PD used are directly interpretable as parameter probabilities (Makowski et al., 2019a). Moreover, the BFs computed for this retrospective hypothesis evaluation provided evidence in line with the interpretation that the null findings may reflect a genuine absence of effect. Because both BFs and the model estimates are sensitive to prior specification (Kruschke, 2014), weakly informative priors have been employed in this retrospective analysis, treating them primarily as a regularization method for the inference (Simpson et al., 2015).

In the context of an underpowered sample size, as in the present study, failing to reject the null hypothesis under the NHST framework, given the error-rate characteristics imposed by the design and practical constraints, leaves open the question of whether the result reflects absence of evidence or evidence of absence. Crucially, a retrospective Bayesian analysis can help disentangle these two scenarios (Dienes, 2014), provided that this interpretative advantage is approached with caution: Bayesian conclusions depend on the choice of priors, which could be weakly informative to encode plausible effect sizes (Simpson et al., 2015), but should not be formed (and informed) to generate evidence that is not present in the data. While BFs and posterior summaries can help clarify whether null results constitute genuine evidence for no effect, they do so within the bounds set by both the limited data and the selected priors.

Moreover, it is important to recognize that moving from NHST to Bayesian inference involves not merely a change in statistical technique; rather, a shift in the underlying epistemological framework. Adopting a Bayesian approach implies a different perspective on what statistical evidence represents and the types of questions that can be addressed. In NHST, the data are conceptualized as a single realization from a hypothetical sampling distribution with known distributional characteristics. NHST therefore answers the question: “Assuming the null hypothesis is true, how likely are data at least as extreme as those observed?” (Wasserstein and Lazar, 2016). This framework inherently promotes a dichotomous outcome: reject or fail to reject the null hypothesis. In contrast, Bayesian uses posterior probability distributions to express the uncertainty associated with model parameters (Greenland, 2006), using the observed data and the prior. Bayesian analysis can therefore answer questions such as: “Given the observed data, the model, and the priors, how probable is it that the parameter is positive, negative, or near zero?” Rather than producing a binary decision, it provides a posterior probability distribution over the parameter space. Thus, differences in interpretive richness between NHST and Bayesian inference arise not from the inherent superiority of one method over the other, but from their distinct conceptual foundations and the inferential questions they are intended to address. Using Bayesian analysis to address the same question as NHST, when the Bayesian model is properly specified, does not—and should not—be interpreted as a way to ‘rescue’ non-significant NHST results. Furthermore, when researchers must ultimately decide whether an effect exists and impose decision thresholds, error rates are inevitably brought into play, regardless of the inferential framework adopted (e.g., Kelter, 2021). Researchers should therefore consider which framework best aligns with their research aims and the types of questions they intend to answer. If the goal is to control error rates in confirmatory testing with predetermined criteria for determining whether an effect exists, NHST may be appropriate. If the goal is to estimate effects expressing their uncertainty, incorporate prior information, or evaluate the strength of evidence for competing models, Bayesian methods may be better suited. The choice of framework shapes not only the statistical procedure, but also the interpretive lens through which results are understood and communicated.

### 4.1 Preregistration declaration

The original study was preregistered prior to data analysis on the Open Science Framework (OSF). The preregistration includes research questions, hypotheses, experimental design, sample size determination, inclusion and exclusion criteria, variables, and planned statistical analyses. The preregistration is publicly available at: 10.17605/OSF.IO/RXK6V.

## Data Availability

The original contributions presented in the study are included in the article/Supplementary material, further inquiries can be directed to the corresponding author. The data used in this study is also publicly available and can be accessed through the OSF repository: https://osf.io/ndcpf.

## Footnotes

1 For additional information on the procedure and materials please refer to the OSF pre-registered project.

2 By default, the *brms* package (Bürkner, 2017, 2018) runs four MCMC chains; we decided to draw 3,000 post–warm-up samples from each chain to ensure reliable parameter estimates.

3 Importantly, BFs are interpreted as measures of relative evidence between the models under comparison. Recent methodological work (e.g., Tendeiro et al., 2024) emphasizes that BFs must be interpreted with caution and within the bounds of what the metric represents and should not be conflated with posterior odds or interpreted as direct measures of probability about parameter values.
