# Supplementary material for: Null hypothesis significance testing vs. Bayesian inference using generalized linear mixed models with binary outcomes: a case study under practical design constraints
Source: Front Psychol. 2026 Apr 16;17:1770212. doi: 10.3389/fpsyg.2026.1770212 (PMC13128418; doi:10.3389/fpsyg.2026.1770212)
Supplement: Supplementary file 1 [file Supplementary_file_1.pdf]

APPENDIX

Glossary

Logistic mixed-effects model. A logistic mixed-effects model is a regression model used for binary outcome variables, in which the probability of an event is modeled using a logistic (logit) link function. The model includes fixed effects, representing predictors of interest, and random effects, which account for dependency in the data (i.e., repeated measurements within participants). In the present study, the model was specified as:

$$logit[\Pr(y_{ij}=1)] = \beta_0 + \beta_1 \text{ Delay}_{ij} + \beta_2 \text{ Pressure}_{ij} + \beta_3 \text{ Order1}_{ij} + \beta_4 \text{ Order2}_{ij} + \beta_5 (\text{Delay} \times \text{Order1})_{ij} + \beta_6 (\text{Pressure} \times \text{Order1})_{ij} + \beta_7 (\text{Delay} \times \text{Order2})_{ij} + \beta_8 (\text{Pressure} \times \text{Order2})_{ij} + u_{0j}$$

Here, logit denotes the logistic link function. The parameter β_0 represents the intercept, β_1 and β_2 parameters represent the effect of experimental condition, β_3 and β_4 parameters represent the effect of trial order, and β_5 through β_8 parameters represent the interaction between condition and order. The term u_{0j} denotes a participant-specific random intercept.

Confidence interval (NHST metric). In NHST, an interval constructed by a procedure so that, under repeated sampling, a specified proportion (e.g., 95%) of such intervals would contain the true parameter value. It does not represent the probability about the parameter itself.

Credible interval (Bayesian metric). An interval that directly represents uncertainty about a parameter. A 95% credible interval contains 95% of the posterior probability mass for that parameter.

P-value (NHST metric). The probability, assuming the null hypothesis, of observing a test statistic as extreme or more than the observed one.

Probability of direction (Bayesian metric). A statistic representing the posterior probability that a parameter is either strictly positive or strictly negative, thus providing a measure of effect direction certainty.

Conditional R^2. The proportion of variance explained by both fixed and random effects combined.

Marginal R^2. The proportion of variance explained by the fixed effects.

Intraclass Correlation Coefficient. The proportion of total variance explained by the grouping structure (random effects), which quantify how similar observations are within the same cluster.

ROPE (Region of Practical Equivalence; Bayesian metric). A predefined range of parameter values considered practically equivalent to no effect. Posterior mass inside the ROPE suggests the effect is negligible in practical terms.

HDI (Highest Density Interval; Bayesian metric). The narrowest interval containing a specified proportion (e.g., 95%) of the posterior distribution, such that all values inside the interval have higher posterior density than those outside.

P-MAP (Bayesian metric). The Bayesian analogue of the p-value, defined as the ratio of the posterior density at 0 to the density at the Maximum A Posteriori estimate.

Akaike Information Criterion (used as NHST metric). A metric which constitute an estimator of prediction error and by which the relative quality of different models can be compared.

Bayes Factor (Bayesian metric). A ratio of marginal likelihoods of two competing models. Bayes factors quantify relative evidence between models, but do not convey parameter uncertainty.
